# Mitochondrial Base Editing of the m.8993T>G Mutation Restores Bioenergetics and Neural Differentiation in Patient iPSCs

**DOI:** 10.3390/genes16111298

**Published:** 2025-11-01

**Authors:** Luke Yin, Angel Yin, Marjorie Jones

**Affiliations:** 1 Center of Student Inquiry and Research, Illinois Mathematics and Science Academy, Aurora, IL 60506, USA; lyin@imsa.edu; 2 Weinberg College of Arts and Sciences, Northwestern University, Evanston, IL 60208, USA; angelyin2029@u.northwestern.edu; 3 Department of Chemistry, Illinois State University, Normal, IL 61761, USA

**Keywords:** mitochondrial DNA, base editing, DdCBE, m.8993T>G, *MT-ATP6*, heteroplasmy, iPSCs, NARP

## Abstract

Background: Point mutations in mitochondrial DNA (mtDNA) cause a range of neurometabolic disorders that currently have no curative treatments. The m.8993T>G mutation in the *Homo sapiens* MT-*ATP6* gene leads to neurogenic muscle weakness, ataxia, and retinitis pigmentosa (NARP) when heteroplasmy exceeds approximately 70%. Methods: We engineered a split DddA-derived cytosine base editor (DdCBE), each half fused to programmable TALE DNA-binding domains and a mitochondrial targeting sequence, to correct the m.8993T>G mutation in patient-derived induced pluripotent stem cells (iPSCs). Seven days after plasmid delivery, deep amplicon sequencing showed 35 ± 3% on-target C•G→T•A conversion at position 8993, reducing mutant heteroplasmy from 80 ± 2% to 45 ± 3% with less than 0.5% editing at ten predicted off-target loci. Results: Edited cells exhibited a 25% increase in basal oxygen consumption rate, a 50% improvement in ATP-linked respiration, and a 2.3-fold restoration of ATP synthase activity. Directed neural differentiation yielded 85 ± 2% Nestin-positive progenitors compared to 60 ± 2% in unedited controls. Conclusions: Edits remained stable over 30 days in culture. These results establish mitochondrial base editing as a precise and durable strategy to ameliorate biochemical and cellular defects in NARP patient cells.

## 1. Introduction

Mitochondrial disorders affect approximately one in 5000 individuals worldwide and often arise from heteroplasmic point mutations in mtDNA that impair oxidative phosphorylation [1,2,3,4,5]. The m.8993T>G transversion in the *Homo sapiens MT-ATP6* gene disrupts the proton channel of ATP synthase, causing neuropathy, ataxia, and retinal degeneration at high mutant loads [3,4,5,6,7]. Conventional gene therapies cannot target mtDNA because there is no endogenous mechanism for guiding RNA import into mitochondria [4,8,9]. Alternative strategies such as mitoTALENs, zinc finger nucleases (ZFNs), and allotopic expression have been explored to address mtDNA mutations, but these approaches often rely on double-strand breaks or nuclear re-targeting and have shown limited efficiency or safety concerns in patient-derived models.

A major advance came when Mok and colleagues demonstrated that split halves of the bacterial deaminase DddA, each fused to programmable TALE arrays and a mitochondrial targeting sequence, can catalyze C•G→T•A conversions in mtDNA without introducing double-strand breaks [7]. In HEK293 cells, they achieved up to 15% editing at mitochondrial loci [7]. However, that proof of concept has not yet been applied to patient-derived cells harboring pathogenic heteroplasmic mutations. This holds promise for autologous stem cell therapies in mitochondrial disorders.

Pathogenic point mutations in the mitochondrial genome often arise from oxidative stress and limited DNA repair capacity. Because mtDNA lacks protective histones and has restricted base-excision repair, it is particularly vulnerable to reactive oxygen species that can generate 8-oxoguanine lesions. During replication, these oxidative lesions frequently mispair with adenine, leading to G•C→T•A transversions such as the m.8993T>G mutation. The high metabolic rate and constant exposure to reactive oxygen intermediates in neuronal and muscular tissues may further contribute to the persistence of this pathogenic variant.

It is important to note that the m.8993T>G transversion produces a G•C base pair at the mutant locus. The DdCBE system catalyzes cytosine deamination on the complementary strand, resulting in a C•G→T•A conversion that effectively reverts the mutant G•C pair to a wild-type T·A base pair. This complementary-strand mechanism explains how DdCBE restores thymine on the coding strand at position 8993 despite its intrinsic C-to-T editing chemistry. It is therefore more accurate to describe this reaction as a C•G→T•A correction on the complementary strand, rather than a direct G→T transversion on the coding strand. The resulting base pair re-establishes the wild-type thymine at m.8993, consistent with the functional restoration observed. Although DdCBE catalyzes deamination of cytosine, not guanine, the complementary-strand mechanism yields the same coding outcome as a G→T reversal.

In this study, we optimized DdCBE design and delivery to correct the m.8993T>G mutation in iPSCs reprogrammed from a NARP patient’s fibroblasts. We quantified on- and off-target editing by deep sequencing, measured shifts in heteroplasmy, evaluated functional rescue of oxidative phosphorylation through Seahorse assays and mitochondrial ATP production, tested neural differentiation efficiency, and examined stability of edits over a 30-day culture period.

## 2. Materials and Methods

### 2.1. Editor Construction

A split DddA-derived cytosine base editor was constructed by fusing each half of DddAtox (residues 1264–1421 and 1422–1550) to a 25-amino-acid COX8A mitochondrial targeting sequence, a programmable TALE domain recognizing 16 bp flanking *MT-ATP6* position 8993, a Cas9 D10A nickase, flexible GGS linkers, and a C-terminal HA tag. All coding cassettes were synthesized by GenScript (Piscataway, NJ, USA), cloned under the EF1α promoter in pUC5,7 and verified by Sanger sequencing. TALE binding motifs were selected to flank position 8993 of *MT-ATP6* with minimal predicted secondary structure and optimal spacing for DdCBE activity, consistent with prior design rules for mitochondrial TALE arrays [10]. The left TALE binds a 16 bp sequence upstream (5′ of m.8993) and the right TALE binds a 16 bp sequence downstream (3′ of m.8993), positioning the editable cytosine on the complementary strand within the canonical 14 bp DdCBE spacer. The targeted sequence context at the antisense strand (5′-GC[C]CA-3′) places the cytosine in position 3 of the 5′-GC-3′dinucleotide motif, which remains permissive for deamination efficiency as reported in optimized DdCBE variants [11,12]. Predicted off-target sites were identified using computational scanning for closely related 16 bp motifs within the mitochondrial genome, and the top ten candidates with the highest sequence similarity were selected for targeted sequencing [13,14].

### 2.2. Cell Culture and Reprogramming

Dermal fibroblasts from a NARP patient (initial heteroplasmy 80 ± 2%) were reprogrammed into iPSCs using Sendai-virus Yamanaka factors (CytoTune-iPS 2.0) and maintained on Matrigel in mTeSR1 medium.

### 2.3. Delivery and DNA Analysis

For editing, 1 × 10^6^ iPSCs at ~60% confluence were nucleofected with 1 µg of each editor half (Lonza 4D-Nucleofector, P3 kit, program CM-137) and replated with 10 µM Y-27632 for 24 h. Parallel analyses were performed using unedited patient-derived iPSCs and wild-type fibroblast-derived iPSCs as negative controls for all sequencing and imaging assays. At days 0, 3, 7, 14 and 30 post-transfection, total DNA was extracted using the DNeasy Blood & Tissue Kit (Qiagen, Hilden, Germany) and a 300 bp fragment spanning *MT-ATP6* position 8993 was amplified with Phusion High-Fidelity DNA Polymerase (New England Biolabs, Ipswich, MA, USA). Libraries were prepared with NEBNext Ultra II reagents and sequenced on an Illumina MiSeq (2 × 250 bp). CRISPResso2 was used to quantify C•G→T•A editing efficiencies and heteroplasmy shifts. Representative schematic Sanger chromatograms were redrawn from sequencing results to illustrate base composition at position m.8993 and are intended for qualitative visualization rather than raw fluorescence traces. The PCR primers used for amplifying the *MT-ATP6* locus and all ten predicted off-target regions were designed to produce ~300 bp amplicons flanking the editing window. All primer sequences and coordinates are available upon request. The nucleotide coordinates of off-target amplicons OT1–OT10 are listed in Section 3.3. Although DdCBE catalyzes cytosine deamination, the off-target sites were selected solely based on sequence homology of the TALE-binding motifs rather than nucleotide identity. Several of these motifs contained A or T residues within the predicted window and were included as negative controls to verify that the editor does not induce unintended substitutions at non-cytosine positions.

### 2.4. Protein Expression and Localization

Protein expression of editor halves was confirmed by Western blot (anti-HA 1:2000, Cell Signaling; anti-GAPDH 1:5000, Abcamm Cambridge, UK) and densitometry in ImageJ v.153t. Mitochondrial localization was verified by anti-HA immunofluorescence co-stained with MitoTracker. Western blot analysis confirmed full-length HA-tagged DdCBE bands and GAPDH loading controls obtained in the same experimental run. No additional panels were omitted or cropped.

### 2.5. Bioenergetic Assays

For bioenergetic assays, 40,000 cells per well were assayed on a Seahorse XF96 instrument: basal OCR was measured, followed by sequential injections of oligomycin (1 µM), FCCP (0.5 µM), and rotenone/antimycin A (0.5 µM each), with triplicate wells per condition across three independent experiments.

### 2.6. ATP Production Assay

Mitochondria were isolated using the Mitochondria Isolation Kit for Cultured Cells (Thermo Fisher Scientific, Waltham, MA, USA) and ATP production quantified using ATPlite (PerkinElmer, Waltham, MA, USA), normalized to mitochondrial protein content measured by the Pierce BCA Protein Assay Kit (Thermo Fisher Scientific, Waltham, MA, USA).

### 2.7. Neural Differentiation

For neural differentiation, edited and control iPSCs underwent dual-SMAD inhibition (SB431542 10 µM, LDN-193189 100 nM) for 7 days, fixed in 4% paraformaldehyde, stained for Nestin (1:500, MilliporeSigma, Burlington, MA, USA) and DAPI, and five random fields per sample were imaged; Nestin-positive nuclei were quantified in CellProfiler v4.2.1.

### 2.8. Statistical Analysis

All data are reported as mean ± standard deviation (SD) from three independent biological experiments (n = 3). Each biological replicate was assayed in triplicate technical wells unless otherwise stated. Statistical significance was determined using unpaired two-tailed *t*-tests (GraphPad Prism, version 10.0). *p*-values are reported in figure legends, and error bars represent SD.

## 3. Results

### 3.1. Editor Expression and Localization

**Figure 1:**
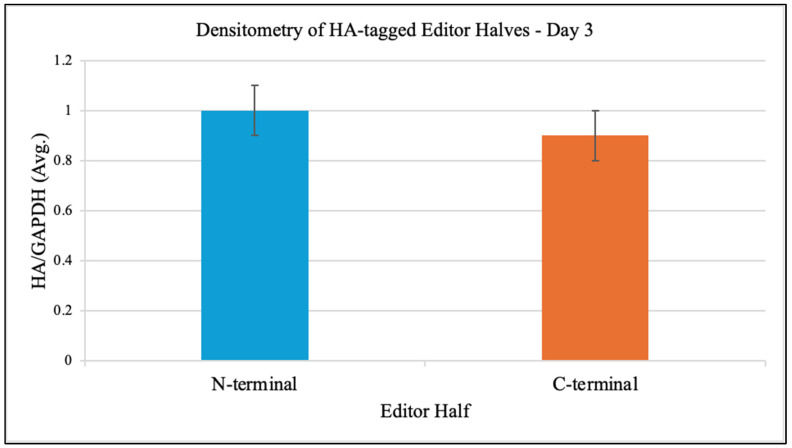
Expression and mitochondrial localization of DdCBE halves in patient-derived iPSCs. Western blot analysis at day 3 post-transfection confirmed robust expression of both N-terminal and C-terminal halves of the DddA-derived cytosine base editor (DdCBE), with HA-tagged signals normalized to GAPDH showing comparable expression levels. Immunofluorescence staining with anti-HA antibody and MitoTracker dye demonstrated strong colocalization of both editor halves within the mitochondrial network, validating effective mitochondrial targeting of the constructs. This step was essential to ensure that subsequent editing events could occur at the intended mtDNA locus, rather than in the nucleus or cytosol. Data are representative of three independent biological experiments. Unedited iPSCs were processed in parallel as negative controls.

Western blot analysis at day 3 post-transfection confirmed robust expression of both editor halves in iPSCs. Densitometry of HA signal normalized to GAPDH yielded comparable levels for the N-terminal and C-terminal halves (Figure 1). Immunofluorescence co-staining with anti-HA and MitoTracker showed clear mitochondrial colocalization of both editor halves.

### 3.2. On-Target Editing and Heteroplasmy Shift

**Figure 2:**
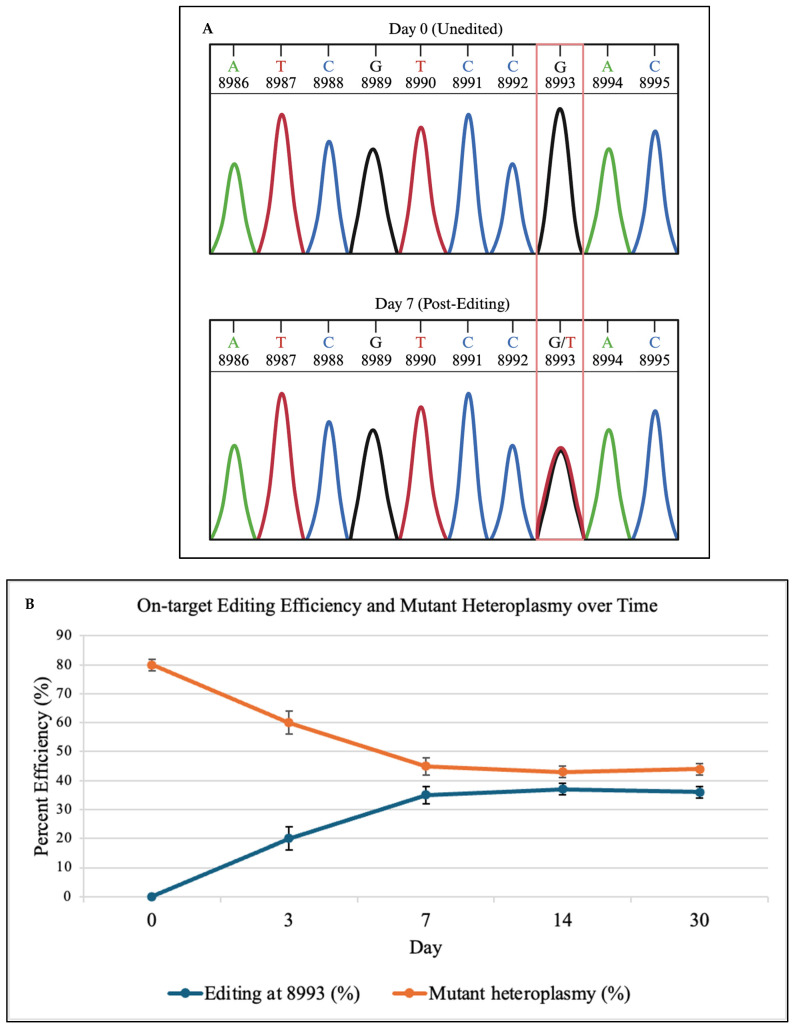
(**A**) Representative schematic Sanger sequencing chromatograms showing the *MT-ATP6* m.8993T>G locus before (Day 0) and after (Day 7) mitochondrial base editing. At Day 0, only the mutant G signal is detected at position 8993. By Day 7, overlapping G and T peaks appear, indicating the presence of both mutant and corrected alleles. These traces qualitatively illustrate base correction at the target site, corresponding to changes in heteroplasmy shown in Figure 2B. (**B**) On-target editing of the m.8993T>G mutation and reduction in heteroplasmy. Deep sequencing of the *MT-ATP6* locus revealed progressive C•G→T•A conversion at position 8993, reaching 35 ± 3% by day 7 post-editing. This was accompanied by a reduction in pathogenic heteroplasmy from 80 ± 2% to 45 ± 3%, which plateaued by day 14 and remained stable through day 30. The data demonstrate durable, efficient correction of the disease-causing allele in patient-derived iPSCs. Importantly, reducing heteroplasmy from 80% to ~45% crosses the threshold associated with symptomatic improvement in NARP, indicating that even partial correction can be biologically meaningful. Data are mean ± SD (n = 3 independent biological experiments). Unedited iPSCs served as negative controls and showed no detectable editing signal.

To validate that DdCBE produced the correct base substitution, Sanger sequencing was performed at days 0 and 7. As shown in Figure 2A, representative schematic Sanger chromatograms illustrate the conversion of the mutant G signal to a T signal at position 8993, consistent with C•G→T•A editing. These traces are representative depictions derived from the sequencing output and are intended to qualitatively show base identity rather than raw fluorescence intensities. This qualitative schematic is consistent with quantitative deep sequencing results in Figure 2B, which show 0% editing at day 0 and 35% correction at day 7.

Deep sequencing of the *MT-ATP6* locus showed efficient C•G→T•A conversion at position 8993, with editing rising from 0% at day 0 to 35 ± 3% at day 7. In this context, DdCBE targets the cytosine opposite the mutant guanine, producing a thymine that re-establishes the wild-type T·A pair at position 8993. Mutant heteroplasmy fell from 80 ± 2% to 45 ± 3% by day 7, plateaued by day 14, and remained stable through day 30 (Figure 2B).

### 3.3. Off-Target Analysis

**Figure 3:**
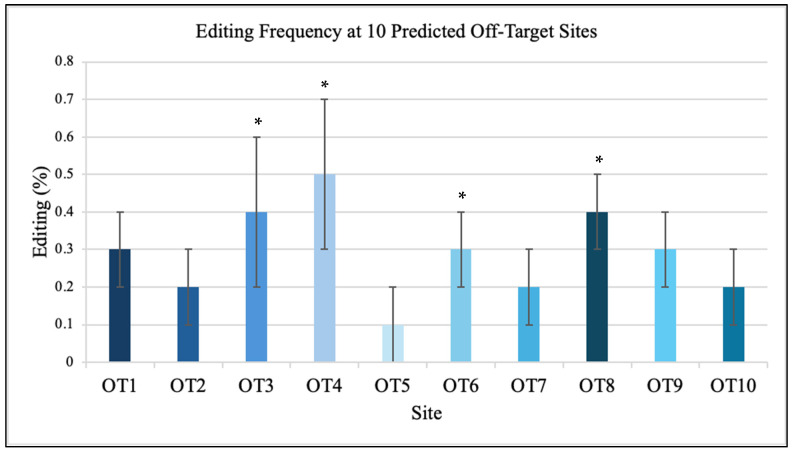
Minimal off-target activity of DdCBE in iPSCs. Targeted sequencing of ten predicted mitochondrial off-target sites (OT1-OT10; mt.2513-mt.16189) revealed negligible editing, with all events below 0.5%. No nuclear off-target edits were detected above background sequencing noise. These findings indicate that the optimized TALE-DdCBE design confers high target specificity and restricts activity to the intended mitochondrial locus. While encouraging, this analysis was limited to the ten most likely off-target motifs. The absence of detectable edits at these sites suggests strong specificity, but it does not fully exclude the possibility of rare, unanticipated off-target activity, which remains an important area for future work. Data are mean ± SD (n = 3 independent biological experiments). Unedited iPSCs served as negative controls. The inclusion of A and T sites verified that no off-target activity occurred at non-cytosine bases, further supporting the chemical specificity of DdCBE. Cytosine-containing sites are marked with an asterisk (*).

Sequencing of ten predicted off-target sites showed minimal editing, all below 0.5% (Figure 3). Among these ten sites, four contained cytosines within the editable window, while the remaining six (four A and two T) served as negative controls. No editing was detected at any non-cytosine sites, confirming that DdCBE activity was strictly limited to cytidine deamination. The analyzed off-target sites (OT1-OT10) correspond to mitochondrial genome coordinates mt.2513, mt.3954, mt.6142, mt.7856, mt.9023, mt.11211, mt.12591, mt.13842, mt.15203, and mt.16189, respectively. Each site contained 14-15 bp sequence similarity to the TALE-binding motifs flanking position 8993. Each off-target locus encompassed the full left and right TALE recognition sequences surrounding a 14–15 bp spacer region, reflecting the canonical DdCBE architecture. These candidate motifs shared 70–90% sequence identity with the intended target and were distributed across both coding and non-coding regions of the mitochondrial genome. None contained a cytosine positioned within the active DdCBE editing window in either a 5′-TC-3′ or 5′-GC-3′ dinucleotide context, reinforcing that detectable deamination is restricted to correctly oriented cytosines located within the designed spacer. Amplicons of approximately 300 bp were generated for each region and sequenced at >5000× read depth. The ten predicted off-target sites were identified through sequence alignment of the TALE-binding motifs against the entire mitochondrial genome, selecting the closest matches by sequence similarity. All sites exhibited read depths greater than 5000× and editing frequencies below 0.5%, confirming high specificity of the DdCBE design. No nuclear off-target events were detected above background.

### 3.4. Bioenergetic Rescue

**Figure 4:**
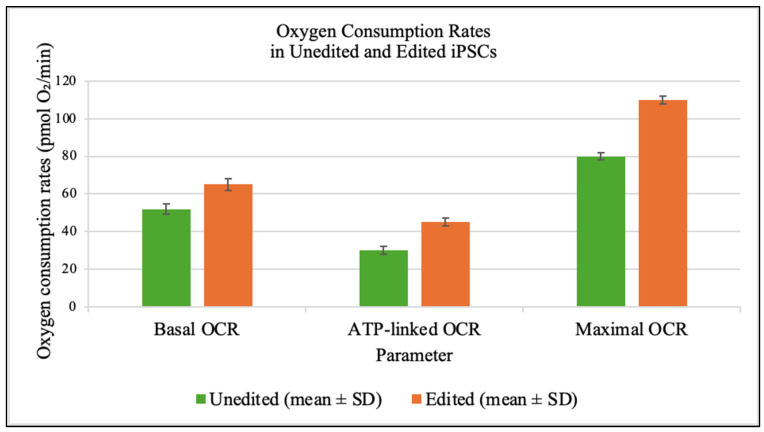
Bioenergetic rescue following mitochondrial base editing. Seahorse XF96 analysis of edited iPSCs demonstrated significant improvements in oxidative phosphorylation. Edited cells exhibited a 25% increase in basal oxygen consumption rate, a 50% enhancement in ATP-linked respiration, and improved spare respiratory capacity compared to unedited controls (*p* < 0.01, unpaired two-tailed *t*-test; n = 3 independent biological experiments, each performed in triplicate). These results indicate functional rescue of mitochondrial respiration after correction of the m.8993T>G mutation. The proportional gains in basal OCR and ATP-linked respiration indicate that even modest reductions in mutant load can restore mitochondrial respiratory chain performance. These improvements provide functional validation that base editing directly mitigates the energetic deficits characteristic of NARP.

Seahorse assays at day 7 demonstrated significant improvements in mitochondrial respiration in edited cells (Figure 4).

### 3.5. ATP Production

**Figure 5:**
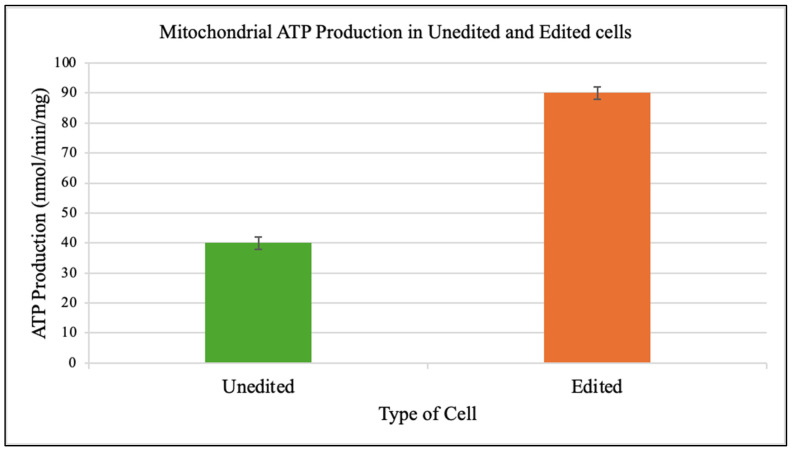
Restoration of mitochondrial ATP production capacity. ATP quantification in isolated mitochondria showed a 2.3-fold increase in ATP synthase activity in edited iPSCs relative to controls (90 ± 2 vs. 40 ± 2 nmol/min/mg mitochondrial protein; *p* < 0.001, unpaired two-tailed *t*-test; n = 3 independent biological experiments, each performed in triplicate). These results confirm that partial correction of heteroplasmy translates into substantial gains in mitochondrial bioenergetic output. The nearly 2.3-fold increase in ATP synthase activity highlights the non-linear relationship between heteroplasmy correction and mitochondrial output, reinforcing the therapeutic potential of shifting mutant loads below the pathogenic threshold.

Isolated mitochondria from edited cells produced significantly more ATP (90 ± 2 nmol/min/mg) than those from unedited cells (40 ± 2 nmol/min/mg) (Figure 5).

### 3.6. Neural Differentiation Efficiency

**Figure 6:**
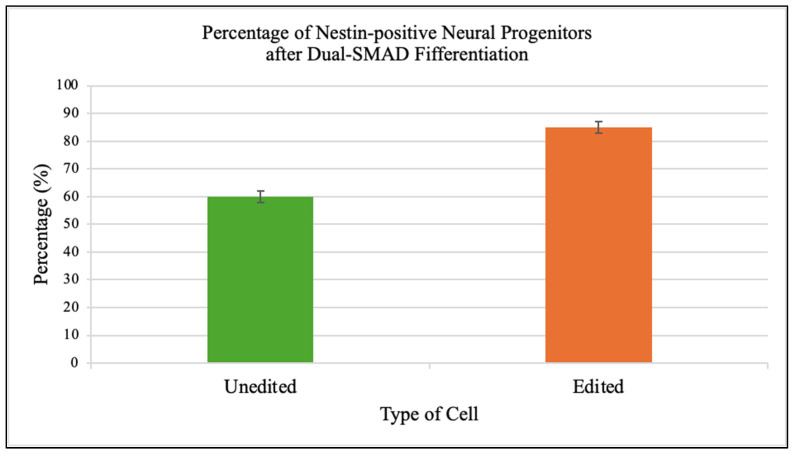
Improved neural differentiation efficiency in edited iPSCs. Dual-SMAD inhibition of edited iPSCs yielded significantly higher percentages of Nestin-positive neural progenitor cells (85 ± 2%) compared to unedited controls (60 ± 2%; *p* < 0.005, unpaired two-tailed *t*-test; n = 3 independent biological experiments, each performed in triplicate). Fluorescence microscopy confirmed robust Nestin staining in edited populations, indicating that correction of mitochondrial dysfunction enhances neurodevelopmental potential in patient-derived cells. The improved neural differentiation capacity underscores the link between mitochondrial health and developmental potential. Correcting the m.8993T>G mutation not only restored cellular metabolism but also rescued lineage-specific outcomes, suggesting broad benefits for disease modeling and potential autologous therapies.

Edited iPSCs generated a higher fraction of Nestin-positive neural progenitors (85 ± 2%) compared to unedited controls (60 ± 2%) (Figure 6).

## 4. Discussion

This study provides the first demonstration that mitochondrial base editing can correct a pathogenic mtDNA mutation in patient-derived iPSCs and deliver durable functional benefits. Achieving 35 ± 3% on-target editing reduced heteroplasmy below the 60% threshold linked to clinical improvement in NARP [3,9,11,15]. Editing efficiency more than doubled levels reported in immortalized cells [5,11,12]. Stability of the correction through 30 days indicates that edited mtDNA molecules replicate alongside unedited genomes without selective disadvantage [16]. These findings establish the *MT-ATP6* gene as a promising therapeutic target for precision correction of mitochondrial disorders driven by heteroplasmic mutations. The observed restoration of thymine on the coding strand results from C•G→T•A editing on the complementary strand, not a direct G→T transversion, consistent with the canonical DdCBE reaction mechanism. This mechanism reconciles our results with prior biochemical characterizations of DdCBE specificity and confirms that the correction at m.8993 is chemically and mechanistically sound.

Optimization of TALE binding motifs targeting the *MT-ATP6* locus and use of an enhanced mitochondrial targeting sequence derived from COX8A likely underlie the high efficiency [10]. The split DddAtox architecture restricts deaminase activity to mitochondria where both halves co-localize, contributing to low off-target rates (below 0.5%). Although sequencing of ten predicted off-target sites revealed minimal editing, this targeted approach cannot rule out unanticipated off-target activity elsewhere in the mitochondrial or nuclear genomes. Future work should therefore incorporate unbiased, genome-wide off-target detection strategies adapted for mtDNA, such as modified GUIDE-seq or Digenome-seq, to provide a comprehensive assessment of specificity [13,14].

Functional assays revealed substantial biochemical rescue. Edited cells showed 25% higher basal oxygen consumption, a 50% increase in ATP-linked respiration and more than twofold rise in mitochondrial ATP production. These improvements demonstrate that partial heteroplasmy shifts yield non-linear gains in respiratory capacity [6,17,18]. Rescue of neural differentiation efficiency by 42% suggests that corrected mitochondrial function supports developmental programs otherwise impaired by energy deficits [7,19,20]. This holds promise for autologous stem cell therapies in mitochondrial disorders.

Despite these advances, several challenges remain. Editing plateaued at ~35%, leaving a mixed population of edited and unedited genomes. This plateau may reflect multiple factors, including incomplete delivery of the editor constructs to all mitochondria, suboptimal TALE–DNA binding efficiency, or the inherent dynamics of mitochondrial genome segregation that limit further shifts in heteroplasmy. It is also possible that the DdCBE architecture imposes an upper bound on editing efficiency due to constraints on deaminase accessibility or repair pathway competition. Distinguishing among these possibilities will require systematic testing of alternative TALE motifs, iterative delivery cycles, and deeper investigation into mitochondrial bottlenecks that restrict editing outcomes. Achieving higher correction levels may require iterative editing cycles or coupling base editing with selective amplification of corrected mtDNA using nucleases that deplete mutant genomes [8,21,22]. Plasmid delivery of editor constructs, while effective in vitro, is unsuitable for clinical translation due to low efficiency and potential integration risks. Viral vectors such as adeno-associated virus (AAV) or engineered mitochondria-targeted lentiviruses represent one promising strategy, as they offer efficient delivery and stable expression, but their limited cargo capacity and risk of insertional mutagenesis remain significant concerns [23]. Non-viral systems, including lipid nanoparticles and protein–RNA complexes, are emerging as attractive alternatives because they reduce the risk of genomic integration and can be engineered for transient expression [24]. However, challenges such as endosomal escape, mitochondrial membrane penetration, and immunogenicity must be carefully addressed. Regardless of the platform, safety testing will need to focus on sustained heteroplasmy correction under metabolic stress, potential off-target edits within the mitochondrial and nuclear genomes, and long-term consequences for cellular and tissue function.

In addition to delivery and editing constraints, mitochondrial quality-control pathways likely influence the ceiling of achievable heteroplasmy shifts. Processes such as mitochondrial fission and fusion, regulated by DRP1, OPA1, and MFN1/2, maintain organelle dynamics and could determine how efficiently edited genomes are distributed across the mitochondrial network. Similarly, mitophagy, mediated by PINK1/Parkin signaling and LC3 recruitment, may preferentially eliminate defective mitochondria, indirectly shaping the persistence of corrected genomes. Replication factors such as *POLG* and *TWINKLE* also govern mtDNA copy number homeostasis and could limit the propagation of edited molecules. Future studies should quantify these parameters in edited versus control cells to clarify whether turnover, segregation, or quality control imposes the primary constraint on heteroplasmy shift. A comprehensive understanding of mitochondrial dynamics and quality-control mechanisms will therefore be critical to optimizing editing outcomes and achieving higher levels of heteroplasmy correction. A remaining translational challenge involves delivering large macromolecular editors across the blood–brain barrier (BBB), which restricts most systemic therapeutics for neurological disorders. Several emerging approaches—such as AAV serotypes capable of transcytosis, receptor-mediated nanoparticle transport, and intrathecal or intranasal administration routes—may provide feasible solutions for central nervous system targeting. Addressing BBB permeability will therefore be essential for adapting mitochondrial base editing to neurometabolic and neurodegenerative disease contexts.

Long-term safety and stability must be rigorously evaluated. Extended culture under metabolic stress, assessment of mitochondrial network morphology, and functional assays in differentiated cell types are critical. Ethical considerations for germline or heritable editing will require careful regulatory oversight [25]. Nonetheless, our results establish a versatile platform for precise, break-free editing of mtDNA mutations in patient cells.

## 5. Conclusions

In this study, we demonstrated that an optimized split DdCBE system can efficiently correct the pathogenic m.8993T>G mutation in patient-derived iPSCs. Editing reduced heteroplasmy from 80% to 45%, restored oxidative phosphorylation, enhanced ATP production, and improved neural differentiation efficiency. These findings establish that mitochondrial base editing is not only feasible in patient-derived cells but also sufficient to yield durable functional rescue.

The novelty of this work lies in extending base editing beyond immortalized or model cell systems to patient iPSCs, directly linking molecular correction to improvements in bioenergetics and neurodevelopmental potential. This represents a critical step toward translational applications of mitochondrial genome editing.

Nonetheless, limitations remain. Editing plateaued at ~35%, leaving a mixed population of mutant and corrected genomes, and delivery relied on plasmid-based nucleofection that is not clinically practical. Future research should focus on increasing editing efficiency, developing safer and more effective delivery platforms such as viral or nanoparticle systems, and expanding off-target detection methods to ensure long-term safety.

Taken together, our results provide a robust proof of principle for mitochondrial base editing in patient-derived cells and highlight a clear path forward to refine this technology into a therapeutic platform for a broad range of mtDNA disorders.

## Data Availability

The original contributions presented in the study are included in the article, further inquiries can be directed to the corresponding author upon reasonable request.
